# Investigation of the simulated microgravity impact on heavy metal biosorption by *Saccharomyces cerevisiae*

**DOI:** 10.1002/fsn3.4034

**Published:** 2024-02-16

**Authors:** Maryam Salavatifar, Kianoush Khosravi‐Darani

**Affiliations:** ^1^ Aerospace Research Institute Ministry of Science, Research and Technology Tehran Iran; ^2^ Department of Food Technology Research, National Nutrition and Food Technology Research Institute Shahid Beheshti University of Medical Sciences Tehran Iran

**Keywords:** adsorption kinetic, biosorption, heavy metal, isotherm model, *Saccharomyces cerevisiae*, simulated microgravity

## Abstract

Heavy metals are one of the most dangerous environmental pollutions, and their elimination is one of the health system's priorities. Microorganisms have been introduced as a safe absorber of such pollution and this ability is related to the characteristics of their surface layers. There are reports about some bacteria's increment of cell envelope thickness in space conditions. Therefore, this study investigated SMG effect on heavy metals biosorption using *Saccharomyces (S.) cerevisiae*. Furthermore, the stability of complex, isotherm, and kinetic absorption models has been investigated. The results showed that the SMG positively affected the biosorption of mercury (Hg) 97% and lead (Pb) 72.5% by *S. cerevisiae*. In contrast, it did not affect cadmium (Cd) and arsenic (As) biosorption. In gastrointestinal conditions, Hg, Cd, and As‐yeast complexes were stable, and their biosorption increased. In the case of the Pb‐yeast complex, in simulated gastric exposure, the binding decreased at first but increased again in simulated intestinal exposure in both SMG and normal gravity (NG). The metals' biosorption by yeast followed the pseudo‐second‐order kinetic and the Langmuir isotherm models for all metals (As) matched with Langmuir and Freundlich. The current research results demonstrate that microgravity provides desirable conditions for heavy metal biosorption by *S. cerevisiae*. Furthermore, the biosorbent–heavy metal complex remains stable after simulated gastrointestinal conditions. Altogether, the results of this study could be considered in detoxifying food and beverage industries and maintaining astronauts' health.

## Graphical abstract

Based previous reports which indicates increased biofilm formation of microbes under microgravity, we assumed a probable change in heavy metal biosorption capacity due to similar mechanism and changes in cell wall. Also, stability of binds between yeast and heavy metals were evaluated in gastrointestinal track. The results showed an increased absorption in most heavy metals.

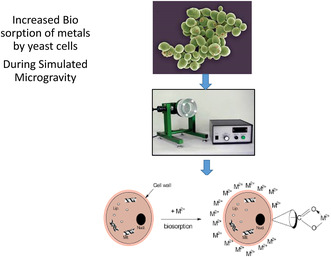

## 1 INTRODUCTION

Many natural elements in the Earth's crust with high atomic densities (above 5 g cm^−3^) are heavy metals (Pourret & Hursthouse, 2019). Agricultural and industrial activities are some of these metal sources that are released into the environment (Bahiru & Yegrem, 2021). Humans and other living organisms are exposed to these elements via oral ingestion, inhalation, and skin contact (Sanaei et al., 2021). Heavy metals, such as mercury (Hg), lead (Pb), cadmium (Cd), and arsenic (As), have irreparable effects on health, even in small amounts (El‐Ghiaty & El‐Kadi, 2023; Seydi & Pourahmad, 2023; Wang et al., 2021). They have acute and chronic toxicity on different body organs. Cardiovascular and nervous system damage, dysfunction of the endocrine glands, immune system, liver, kidney, bones, lungs, and cancer are some consequences of entering and accumulating these metals in the body (Balali‐Mood et al., 2021; Sankhla & Kumar, 2019). Synergistic effects may occur due to concurrent exposure to more than one metal (Singh et al., 2017). World Health Organization (WHO) guidelines establish permissible Hg, Pb, Cd, and As levels in drinking water at 6, 10, 3, and 10 g L^−1^ (Khosravi‐Darani et al., 2021). Due to their high toxicity and destructive effects, removing them is on the research agenda (Feng et al., 2022; Khosravi‐Darani et al., 2021; Zoghi et al., 2022).

A wide range of strategies for heavy metal elimination have been proposed, such as adsorption, filtration, precipitation, coagulation, and ion exchange. However, these methods have faced limitations because of their high energy requirements, restrictions on metal concentration, high cost, and toxic byproducts (Renu, Agarwal, & Singh, 2017). Therefore, for this type of decontamination, a cost‐effective and efficient method, such as biological removal, should be considered (Grujić et al., 2018; Zoghi et al., 2014). Yeast, bacteria, algae, and fungi are some microorganisms that effectively bioremediate heavy metals (Abid et al., 2022). Several studies have demonstrated that *Saccharomyces (S.) cerevisiae* can bind toxic metals in aqueous environments and reduce their bioavailability (Khosravi‐Darani et al., 2021; Wang et al., 2021). *S. cerevisiae* is a promising and safe yeast that grows in a low‐cost medium. It is widely used as a probiotic in fermentation industries, such as food, drinks, and feed supplements (Abid et al., 2022). The metal may cause adverse health effects as it is released from the microorganism during passage through the digestive tract. As a result, binding stability must also be carefully investigated in addition to strength (Zoghi et al., 2014).

Microorganisms have to sense and respond to any alteration in their living environment in order to survive. They have to sense and respond to any alteration in their living environment including gravity. Microorganisms have molecular mechanisms that can sense gravity and respond to it directly or indirectly. Any change in this force may affect their physiology, morphology, and pathogenicity (Nickerson et al., 2004). Several studies have proven that microgravity conditions cause changes in microorganisms that can be used in the fields of genetic engineering and biotechnology (Huangfu et al., 2020; Qi et al., 2011; Salavatifar, Mosallaei, & Salmanian, 2022). Microorganisms’ bioremediation of toxins and heavy metals has also been determined (Afraz et al., 2020; Afsharian et al., 2022; Khosravi‐Darani et al., 2021). Moreover, our previous study has demonstrated that simulated microgravity (SMG) conditions have increased heavy metal bioremediation by *Lactobacillus (L). acidophilus*, but the stability of biosorbent and some heavy metals was not enough in simulated gastrointestinal conditions (Afsharian et al., 2022).

Some studies have investigated the absorption power of heavy metals by yeasts, especially *S. cerevisiae* (Hadiani et al., 2019; Hadiani, Darani, et al., 2018; Hadiani, Khosravi‐Darani, et al., 2018; Mirmahdi et al., 2022; Zinicovscaia et al., 2020). However, to the author's knowledge, no study has been conducted on the effect of microgravity on the heavy metal biosorption by *S. cerevisiae*. In addition, no study has measured the microgravity effect on the strength of the heavy metal–*S. cerevisiae* binding in simulated GIT conditions (Jena et al., 2022).

As a result, the main objectives of this study were gravity impact investigation on Hg, Pb, Cd, and As bioremediation in ppb (parts per billion = μg L^−1^) scale by *S. cerevisiae* from water. In addition, the effect of gravity on the bond stability between *S. cerevisiae* and these metals under simulated gastrointestinal conditions was also investigated. Furthermore, the isotherm and kinetic absorption models have been studied. Through this investigation, we hope to achieve more effective conditions for heavy metal biosorption that are applicable in food industries. Also, the results of this study may be useful for maintaining the health of astronauts.

## 2 MATERIALS AND METHODS

### 2.1 Reagents and chemicals

All culture components and chemical reagents were purchased as analytical purity from Merck (Darmstadt, Germany) except for standard solutions of As (1000 mg L^−1^ in 0.1 M HNO3), which were obtained from Panreac (Panreac Quimica SA, Spain, Barcelona). Deionized water was used to make the solutions. All containers were floated in 15% v.v^−1^ HNO_3_ for 24 h and then rinsed with deionized water to remove metal pollution. To eliminate microbial contamination, containers were autoclaved at a temperature of 121°C and pressure of 1.4 atm for 20 min before starting the experiments.

### 2.2 Simulated microgravity treatment

To simulate weightlessness, a one‐axial clinostat was used (UN00SA, USA). One‐axial clinostats are tools that rotate around an axis perpendicular to the gravity vector at a constant speed, and the gravity vector enters the sample placed on it in different directions. Therefore, the gravitational forces applied to the sample neutralize each other, and weightlessness is simulated. The “microgravity” term refers to the fact that the power of gravity is not entirely equal to zero but is very small and close to zero. The simulation level is determined based on the speed of rotation and the distance of the sample from the center of the device (10^−1^–10^−6^ g) (Dietlein et al., 2013). Because the presence of any bubbles disrupts weightlessness due to the creation of shear stress forces, the tube had to be filled completely (Salim et al., 2010).

### 2.3 Preparation of yeast suspensions

The yeast *S. cerevisiae* ATCC 9763 was supplied from the Research and Technology Department of Ministry of Science, Iran (Persian‐type collection) in freeze‐dried form. Yeast cells were inoculated in a sterilized nutrient broth containing: glucose, 1 g/50 mL distilled water (DW); yeast extract and NH4Cl, 0.25 and 1 g/50 mL DW; and KH2PO4 and Na2HPO4, 0.75 and 1.125 g/50 mL DW. The culture was incubated at 27°C, 80 rpm for 16–20 h (end of the exponential phase). Finally, the prepared master culture was stored at 4°C until bioremoval studies.

Seed culture was prepared daily for each series of bioremoval tests. For preparing the seed culture, 5% (v/v) from the master culture was inoculated to the same medium and shaken at 27°C for 16–20 h. The numbers of *S. cerevisiae* were estimated by direct counting in a Neubauer hemocytometer chamber under an inverted microscope (CETI, UK).

### 2.4 Bioremoval of heavy metals from aqueous solutions using *S. cerevisiae*

An aqueous solution containing AS (95 μg L^−1^), Pb (52.5 μg L^−1^), Cd (52.5 μg L^−1^), and Hg (79.8 μg L^−1^) was prepared. The pH was adjusted to 5 using 0.1 M HCl and 0.1 M NaOH. The basis for choosing heavy metal concentrations was according to the optimization of previous studies (Hadiani et al., 2019; Hadiani, Darani, et al., 2018; Hadiani, Khosravi‐Darani, et al., 2018; Mirmahdi et al., 2022). Then, the 2.5 × 10^8^ CFU mL^−1^ (direct counting) was added to 9 mL of multimetal aqueous solution. These mixtures were placed in a 25°C incubator for 24 h. After filling and bubble removal, microgravity tubes were fixed on a clinostat with a 15 rpm rotational speed (Salavatifar, Mosallaei, & Salmanian, 2022).

### 2.5 Simulated gastric and small intestinal juice preparation and investigation of bond stability

For simulated gastric juice preparation, pepsin (3 g L^−1^) was dissolved in a sterile NaCl solution (0.5% w/v), and the pH was adjusted to 2 with HCl. For the preparation of simulated small intestine juices, pancreatin (1 g L^−1^) and bile salts (1.5 g L^−1^) were added to a sterile NaCl solution (0.5%, w/v). Then, the pH was adjusted to 8 with NaOH. The simulated gastric and small intestinal juice was made daily before use and sterilized with 0.45 μm membrane filters (Nalge, Rochester, NY, USA; Khorasani & Shojaosadati, 2017).

After 24 h of metal bioremoval, a series of samples from both NG and SMG treatments were centrifuged for metal concentration measurement. Another sample series was added to 40 mL of simulated gastric juice and vaporized for 10 s. Then, it was incubated at 37°C for 2 h in NG and SMG conditions. After sampling for metal concentration measurement, 10 mL of every NG and SMG sample was added to 50 mL of simulated intestinal juice. Then, it was incubated at 37°C for 2 h in NG or SMG conditions. Ultimately, sampling for heavy metal measurement was repeated (Yin et al., 2018). Inductively coupled plasma‐mass spectroscopy (ICP‐MS; Perkin Elmer ELAN 6100 DRC‐e, USA) was used in this study for heavy metals concentration measurement.

### 2.6 Adsorption kinetic studies

A predefined count of yeast cells was transferred into water (pH 5.0) containing (μg L^−1^) Hg, Pb, Cd, and AS with 95, 52, 80, and 52 concentrations. Heavy metal solution concentrations were assessed at eight time intervals to study the adsorption kinetics model. As described elsewhere, Temkin, Freundlich, Langmuir, and isotherm models were used to research biosorption isotherms (Zoghi et al., 2021).

### 2.7 Isotherm model studies

Five samples with various numbers of untreated *S. cerevisiae* cells (2.5 × 10^9^ CFU mL^−1^) were mixed with five different initial concentrations of heavy metals (Hg, Pb, Cd, and As; pH 5) for 24 h. Pseudo‐first and pseudo‐second‐order kinetic equations were assessed for heavy metal (Hg, Pb, Cd, and As) adsorption by untreated *S. cerevisiae* strains (Zoghi et al., 2021). Experiments were carried out in triplicate. Parameters of isotherm models were achieved based on the method described (Chen et al., 2015). The sum of error squares regression and coefficient values were used to define the best isotherm model.

### 2.8 Statistical analysis

In this study, all experiments were done in triplicate, and data were presented as mean ± SD (*X* ± SD). The graphs were drawn using GraphPad Prism software (version 9). Results were used in one‐way analysis of variance (ANOVA) to estimate *p*‐values and confidence levels, and significant values less than .05 (*p*‐value < .05) are presented.

## 3 RESULTS AND DISCUSSION

### 3.1 Simulated microgravity effect on heavy metal bioremediation by *S. cerevisiae*

**FIGURE 1:**
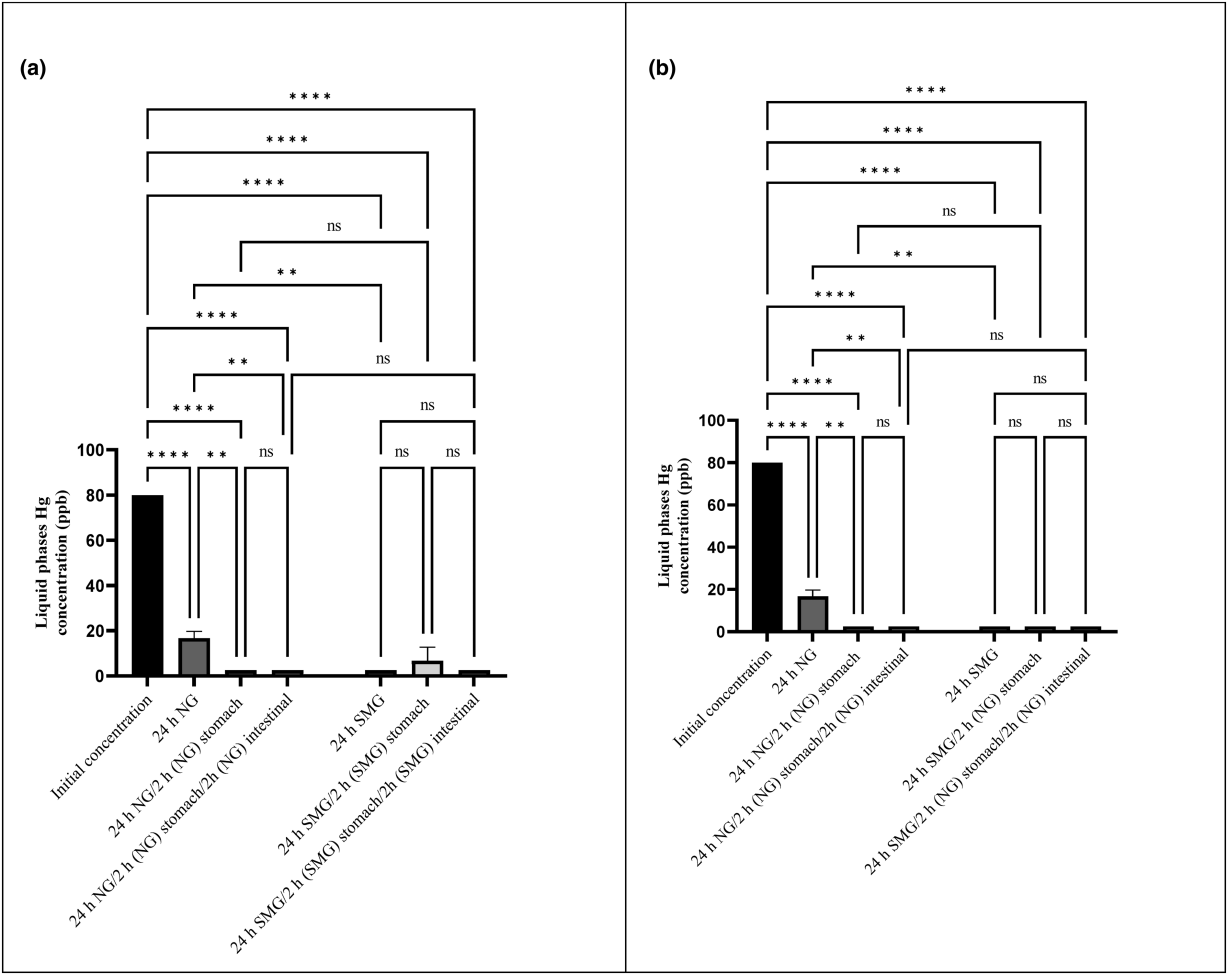
Hg concentrations in liquid phase after 24 h exposure to *Saccharomyces cerevisiae* ATCC 9763 under SMG (a) and NG (b) in gastrointestinal conditions. The standard deviation is considered with 95% confidence (*p*‐value < .05). **p < .01; and ****p < .0001. Ns, nonsignificant.

**FIGURE 2:**
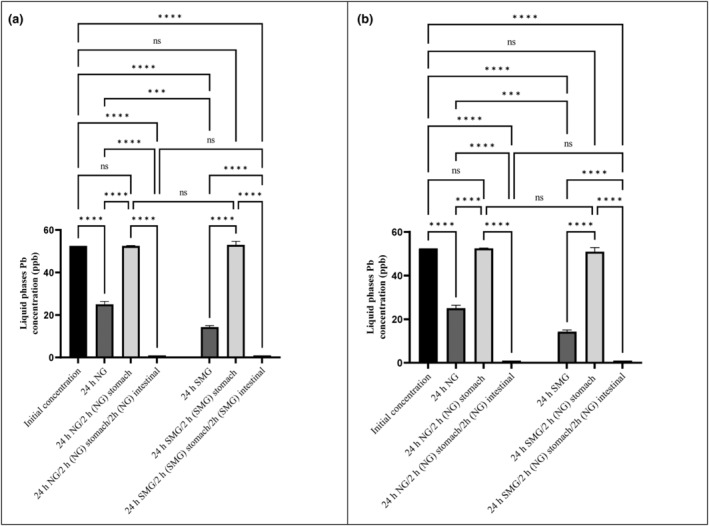
Pb concentrations in liquid phase after 24 h exposure to *Saccharomyces cerevisiae* ATCC 9763 under SMG (a) and NG (b) in gastrointestinal conditions. The standard deviation is considered with 95% confidence (*p*‐value < .05). ***p < .001 and ****p < .0001. Ns, nonsignificant.

**FIGURE 3:**
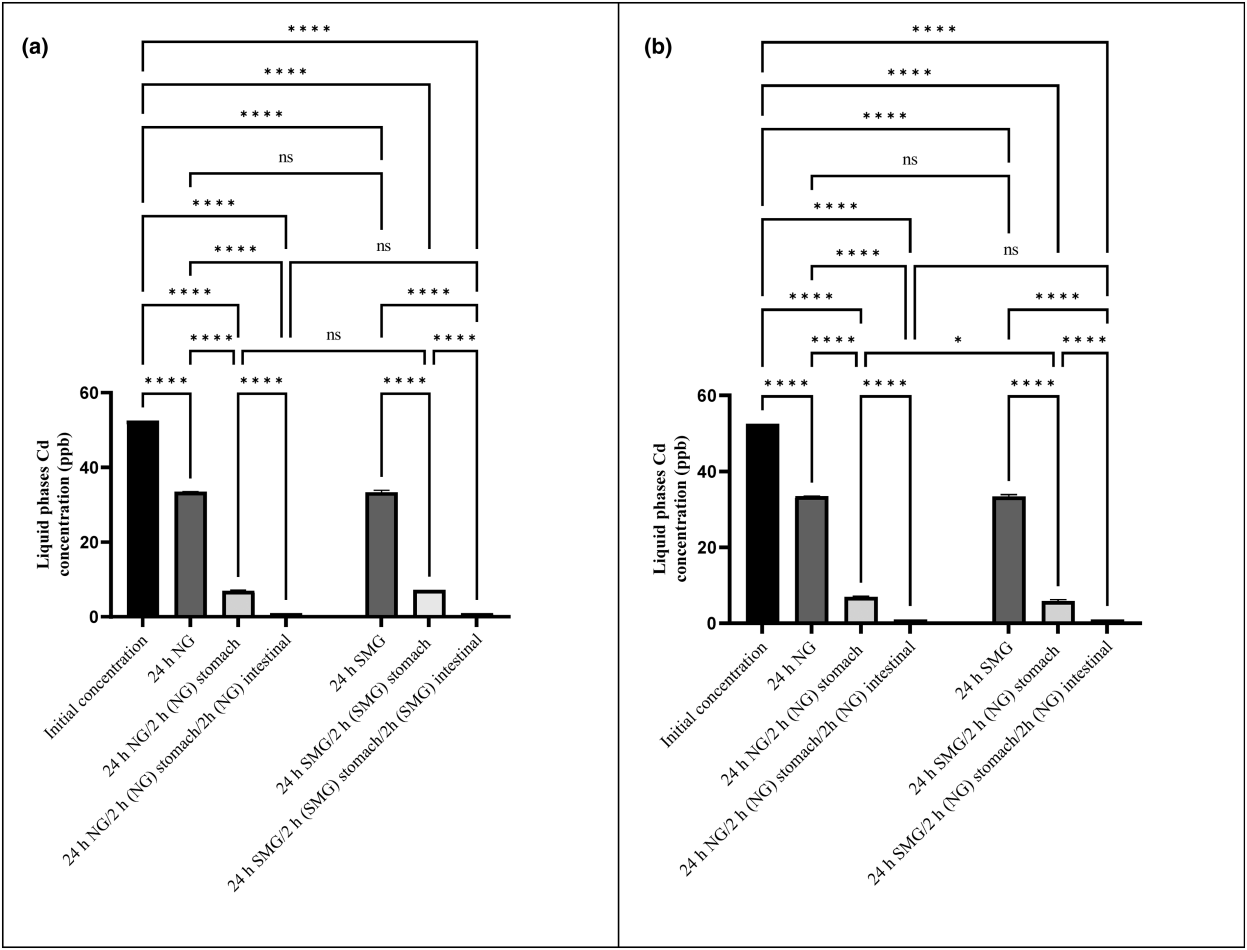
Cd concentrations in liquid phase after 24 h exposure to *Saccharomyces cerevisiae* ATCC 9763 under SMG (a) and NG (b) in gastrointestinal conditions. The standard deviation is considered with 95% confidence (*p*‐value < .05). *p < .05; and ****p < .0001. Ns, nonsignificant.

**FIGURE 4:**
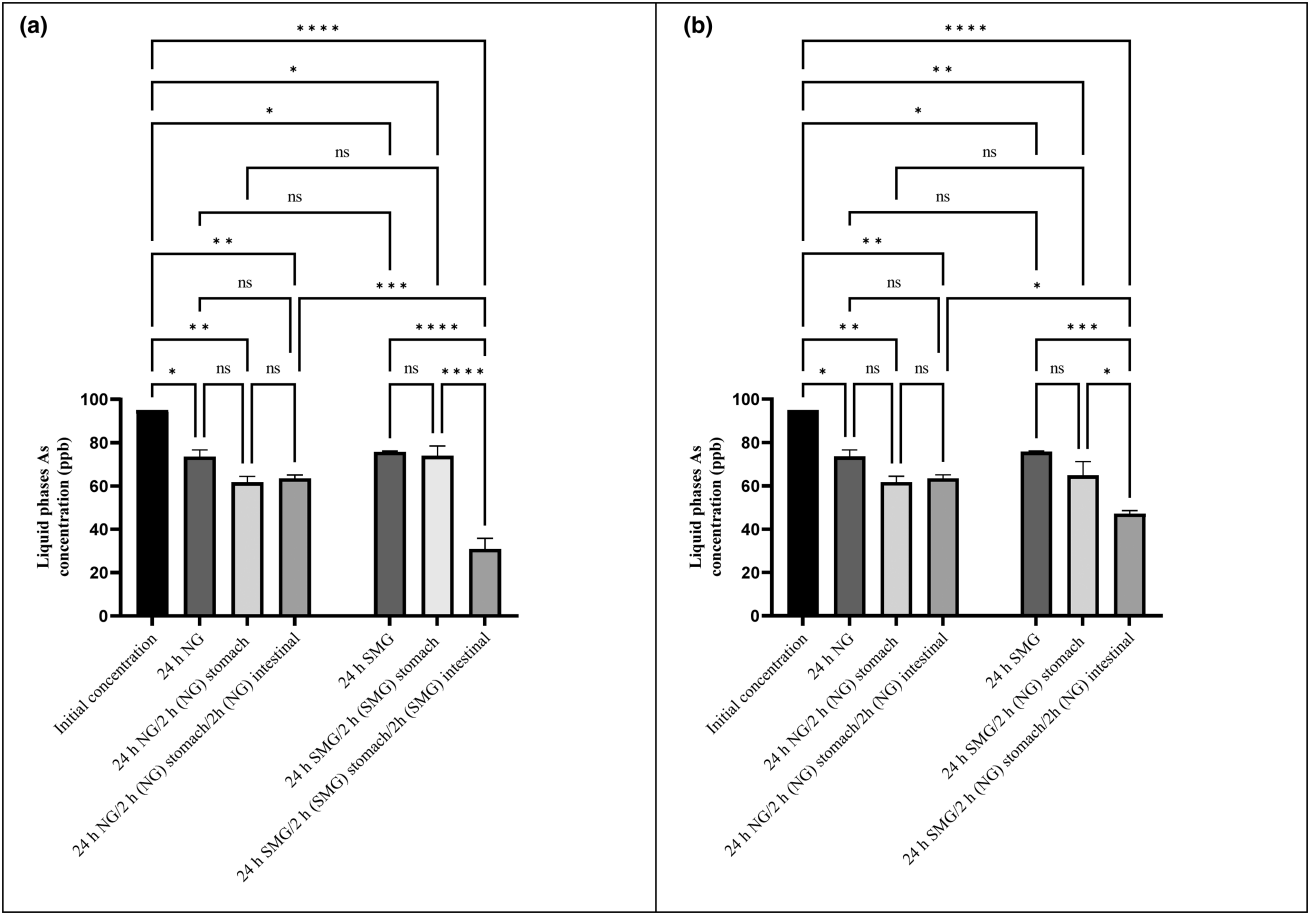
As concentrations in liquid phase after 24 h exposure to *Saccharomyces cerevisiae* ATCC 9763 under SMG (a) and NG (b) in gastrointestinal conditions. The standard deviation is considered with 95% confidence (*p*‐value < .05). *p < .05; **p < .01; ***p < .001; and ****p < .0001. Ns, nonsignificant.

As a result of the present experiment, heavy metal concentrations decreased following 24 h of adjacency to *S. cerevisiae* ATCC 9763 (Figures 1, 2, 3, and 4). For all metals (Hg, Pb, Cd, and As), statistically significant differences were found (*p*‐value < .05). Furthermore, statistically significant differences between SMG and Ng conditions about Hg and Pb were seen. In other words, microgravity positively affected the bioremediation of Hg and Pb by *S. cerevisiae* but had no effect on Cd and As biosorption.

According to comparisons, the bioremediation rate of heavy metals by *S. cerevisiae* from the water followed the order of Hg > Pb > Cd > and As in both MG and NG conditions. This absorption pattern was similar to Mirmahdi et al. (2022) and Afsharian et al. (2022) results about the absorption of metals by S. *cerevisiae* and *L. acidophilus*, respectively. The absorption order of Pb and Cd by S. *cerevisiae* was opposite to Thippeswamy et al. (2014) study in NG, which could be due to the difference in the duration of the adsorbent's adjacent with metals or much higher concentration of metals (Thippeswamy et al., 2014).

As shown in Figure 1, the highest Hg content (97%) was absorbed by *S. cerevisiae* under SMG conditions. While Hg was removed by 79.1% in NG conditions. In Afsharian et al. (2022) study, it was proved that SMG had no significant effect on Hg biosorption by *L*. *acidophilus*. Mirmahdi et al. (2022) show that more Hg (92.7%) was absorbed by S. *cerevisiae* compared to the present study. It may be related to higher amount of inoculum (10 times) or yeast pretreatments.

Based on Figure 2, 72.5% and 52.38% of Pb were removed by *S. cerevisiae* in SMG and NG conditions, respectively (*p*‐value < .05). In contrast, in Afsharian et al. (2022), the Pb removal by *L. acidophilus* was higher in Ng than in SMG. The difference between biosorbent probiotics may explain this conflict.

Figures 3 demonstrated that 36.48% and 36.28% of Cd content had been adsorbed after 24 h of exposure to *S. cerevisiae* in both SMG and NG conditions, respectively. However, there were no significant differences between SMG and NG. The lack of absorption difference between SMG and NG was similar to Afsharian et al. (2022), which was observed with *L. acidophilus* treated with alkaline but not with untreated bacteria.

According to Figure 4, *S. cerevisiae* bioremediation of As was lower than the other metals, and no significant differences were found between SMG and NG (20.3% and 22.61%, respectively). The small amount of As biosorption by *S. cerevisiae* was similar to the previous result (Hadiani et al., 2019; Mirmahdi et al., 2022). The lack of a difference in As biosorption between SMG and Ng was identical to the results of the previous study (Afsharian et al., 2022; Mirmahdi et al., 2022).

Two similar studies conducted by Grujić et al. (2018; 2017) investigated some heavy metals (Zn, Ni, Cd, Hg, Pb, and Cu) impact on the *S. boulardii* biofilm and planktonic cells. The results showed that *S. boulardii* only tolerated Pb and Zn in planktonic cells, not biofilm. It should be noted that the concentrations of metals in the mentioned studies were much higher than in the current study (millimolar range).

A similar study was conducted by Jakovljević et al. (2022) on the absorption of several metals by the biofilm of some individuals and combinations of microorganisms. The results showed that the Pb and Cd absorption by *S. cerevisiae* alone and *K. oxytoca/S. odorifera/S. cerevisiae* combination was above 95% after 10 days (Jakovljević et al., 2022). Therefore, in future studies, the metal biosorption in SMG using biofilm can be investigated.

Because four metals were simultaneously present in the aqueous solution, it can be concluded that bacterial heavy metal bioremoval is selective and dependent on the structure and thickness of the cell wall (Zoghi et al., 2014). The *S. cerevisiae* is one of the promising probiotics for the bioremediation of heavy metals (Mirmahdi et al., 2022). According to microscopic and spectrophotometric observations, bioremediation of metals by yeast is a physical process at their surface (Zinicovscaia et al., 2020). Surface charge is the primary determinant of yeast's ability to bind heavy metals (Senatore et al., 2018). The cell wall of yeast is negatively charged, similar to the other microbes (Nickerson et al., 2004). The phosphate, amino, carboxyl, hydroxyl, and hydrosulfide groups in surface proteins have caused a negative charge and are responsible for binding bivalent metals (Fadel et al., 2017). Furthermore, β‐glucan, which is one of the main components of the yeast cell wall, has a slightly negative charge due to the presence of phosphate groups. So, any factor that increases the thickness of the yeast cell wall, increases the negative charge (Zielke et al., 2018) and thus promotes cationic heavy metal absorption.

As a result of the lack of sedimentation, low shear stress, and slight turbulence of the environment in microgravity, significant changes occur in the characteristics of living organisms (Nickerson et al., 2004). According to Nemoto et al. (2019), reduced gravity can cause changes in gene expression and morphology of *S. cerevisiae* (Nemoto et al., 2019). Furthermore, Liu et al. (2008) demonstrated that spaceflight induced significant changes in cell wall thickness and β‐glucan content of *S. cerevisiae*. In addition, they have also proved that the reduction in glucanase secretion in yeast cells occurs in a microgravity environment (Liu et al., 2008). Glucanases are enzymes that hydrolyze β‐glucan chains (Martín‐Cuadrado et al., 2008). Thus, when the activity of this enzyme is reduced, the content and thickness of the cell wall increase as well (Liu et al., 2008). Increased cell wall thickness increases the cell's ability to bind heavy metals compared to NG on Earth. Laboratory instruments are an excellent way to simulate specific conditions in space. These simulators offer an efficient, cost‐effective, and fast method of research. By using these laboratory tools, scientists can get an understanding of the effects of space travel and its implications on human health. In addition, the results of these studies can be applied to terrestrial applications (Huang et al., 2018). Clinostat is one of these tools to simulate microgravity.

Overall, the results show that microgravity considerably affects the bioremediation of some heavy metals by *S. cerevisiae*. Furthermore, this effect depends on the type of absorbent microorganism and the type of heavy metal.

### 3.2 Stability assessment of *S. cerevisiae*–heavy metal complexes following simulated gastrointestinal exposure

The concentration of all heavy metals after exposure to GIT conditions was presented in Figures 1, 2, 3, 4. The amount of metals after exposure to gastric juice, as well as small intestinal condition, shows the strength of biosorbent‐adsorbed complex. The lower the concentration of metals in this stage, the more potent the metal complex with absorbent. In fact, the stability and strength of the biosorbent‐adsorbed complex under gastrointestinal conditions play an essential role in measuring the efficiency of bioremediation (Ribeiro et al., 2021; Tuzen et al., 2020).

Based on the results of this study, Hg exhibited the highest binding to yeast and the most increased stability of the complex. As shown in Figure 1 following simulated GIT treatment, the strength of the Hg‐yeast complex was highest, and no significant separation of Hg from yeast was observed. It should be noted that a small amount of Hg dissociated from yeast after stomach juice exposure in SMG. However, it was completely removed after exposure to small intestinal conditions in SMG (Figure 1a). In the GIT treatment in NG, no releasement of Hg was observed. Complete removal and strength of the Hg complex with yeast in SMG were similar to Afsharian et al. (2022). Of course, *L. acidophilus* had released some bonded Hg following GIT treatment in NG conditions. The difference may be attributed to the probiotic type and the surface's structure. The results showed that the Pb‐yeast complex was reversible under simulated stomach juice conditions. So, 52.4% and about 72% of the removed Pb were released after simulated gastric exposure in NG and SMG, respectively (Figure 2). However, after simulated small intestinal exposure, bioremoval was significantly enhanced compared with stomach juice and initial concentration in both NG and SMG. The releasement of Pb in gastric juice and rebinding in small intestinal conditions was similar to Mirmahdi et al. (2022) and Zoghi et al. (2022) studies by *L. acidophilus* in NG. As shown in Figure 3, after Cd absorption during the first 24 h, small amount of Cd dissociated from *S. cerevisiae* after stomach juice exposure in SMG and NG. However, it was completely removed after exposure to small intestinal conditions in SMG (significant differences were found).

In fact, SMG did not significantly impact the bioremoval and stability of the cadmium–yeast complex. A similar increase in Cd removal and complex stability was observed in the Mirmahdi et al. (2022). In contrast to the present experiment, SMG positively affected *L. acidophilus* Cd biosorption and stability of the complex (Afsharian et al., 2022). The type of biosorbent used may contribute to inconsistent results.

According to Figure 4, after As absorption, even in a small amount during the first 24 h, the metal–yeast complex remained stable in a simulated gastric exposure in both SMG and NG. However, no changes in absorption were observed. As shown in Figure 4a, no alteration was observed in the amount of As‐yeast complex after exposure to simulated small intestinal juice in NG. Based on this figure, the total amount of As biosorption in the NG condition was 33.5%.

On the contrary, according to Figure 4b, the amounts of totally absorbed As were significantly higher in samples that had spent the first 24 h in SMG (67.5%) than in those that had spent the first 24 h in NG (50.4%). Arsenic concentrations decreased significantly in both cases in comparison with the NG condition. Therefore, removing this metal will benefit space travelers more than consumers on the ground. The comparison of SMG and Ng effects on As removal was similar to that conducted by Afsharian et al. (2022) and Mirmahdi et al. (2022).

The SMG impact on As bioremoval and complex stability of the present study was in accordance with Afsharian et al. (2022).

This release of heavy metal in gastric juice and rebinding in intestinal conditions that was observed mainly in the case of Pb can be related to the acidity of the solution. High hydrogen ions compete with the heavy metal in an acidic environment (similar to stomach juice conditions). Therefore, some of the metal is separated from the adsorbent. However, the competition is lost with the reduction of hydrogen ions in intestinal conditions and metal reattachment occurs. This has been observed by Lecca‐Caballero et al. (2023) in the case of Pb biosorption by *Serratia marcescens* (Lecca‐Caballero et al., 2023). Furthermore, it was suggested that noncovalent electrostatic bonds (e.g., Van der Waals and hydrogen bonds) were formed between heavy metal and yeast (Zoghi et al., 2021).

### 3.3 Kinetic model studies

Kinetic models describe the dependency of biosorption efficiency on the exposure time and mechanism of adsorption of heavy metal by the cell (Tuzen et al., 2020). The results of this study show that biosorption depends on time.

Data of Hg, Pb, Cd, and As adsorption by yeast and correlation coefficient values of R_2_ from the linear regression analysis are shown in Table 1. Kinetic model studies were fitted using pseudo‐first‐order (not shown) and pseudo‐second‐order (Table 1). Pseudo‐second‐order kinetic mechanisms have also described hg bioremoval by *Lactarius acerrimus* (Tuzen et al., 2020) and *Rhizopus oligosporus* (Ozsoy, 2010). Mirmahdi et al. (2022) also showed bioremoval of heavy metal by yeast could be modeled by a second‐order kinetic. Biosorption of metals onto the space of the cell surface might be attributed to intraparticles and particle diffusion (Asare et al., 2018). In different process conditions, including temperature and pH, the kinetic model of metal biosorption had been fitted to pseudo‐second kinetic models. Kinetic studies help to illustrate nature of mechanism of metal biosorption in yeast. Pseudo‐second‐order model showed that metal biosorption is through physic‐chemical interactions.

**TABLE 1:** Kinetic model studies of Hg, Pb, Cd, and As adsorption by yeast: Linear regression analysis of pseudo‐second‐order, as fitted model.

No	Model	Equation	Reference	*R* ^*2*^ correlation coefficient values
1	Pseudo‐first‐order	$\ln\left(q_{e}-q_{t}\right)=\ln q_{e}-K_{1}t$	Al‐Hazmi, (2010)	Not fitted
*K*_1_ is rate constant, *q*_e_ and *q*_t_ adsorbed metals at equilibrium times (*h*), and at a given time (*t*)				
2	Pseudo‐second‐order	$\frac{t}{q_{t}}=\frac{1}{K_{2}q_{e}^{2}}+\frac{t}{q_{e}}$	Anene et al. (2016)	Hg: 0.9991 Pb: 0.9775 Cd: 0.9897 As: 0.9422
*K*_2_ is rate constant, *q*_e_ and *q*_t_ adsorbed metals at equilibrium times (*h*), and at a given time (*t*)				

### 3.4 Biosorption isotherms of heavy metals

Design of adsorption systems on a large scale needs equilibrium data. Associations between cells and liquid phase in equilibrium conditions (10^7^ CFU mL^−1^ of yeast cells at 25°C for 24 h in pH 5) at various initial concentrations of heavy metals could be qualified generally using three different isotherm models of Temkin (1940), Freundlich (1906), and Langmuir (1918). In the biosorption of heavy metal, some of the properties of *S. cerevisiae*, including surface area, functional groups, and structure, play essential roles (Ertugay & Bayhan, 2010).

Hg, Pb, Cd, and As biosorption isotherms are illustrated in Tables 1 and 2. Coefficients of correlation for Pb were calculated as belonging to Temkin, Freundlich, and Langmuir isotherms. The Hg, Pb, Cd, and As bioremediation process was further matched with the Langmuir isotherm model. These data contrasted with Cd biosorption by *Pediococcus pentosaceus* reported by Le and Yang (2019) whose data indicate data fitted to the Freundlich isotherm model (Le & Yang, 2019).

**TABLE 2:** Describe the three mentioned models and regression analysis of the isotherms.

No	Equation	Model	Reference	Regression analysis of the isotherms
1	$Q_{e}=\beta \;\ln\alpha +\beta \;\ln C_{e}$ β=RTKT	Temkin	Temkin (1940)	Hg: 0.9247 Pb: 0.8965 Cd: 0.8387 As: 0.8034
*K*_T_ and *α* are Temkin constant (*R* = 8.314 J/mol.K), *T* is the absolute temperature, *C*_e_ and *Q*_e_ are the equilibrium concentration of metals in aqueous solutions and the number of metals in adsorbing equilibrium.				
2	$Q_{e}=K_{F}\times C_{e}^{1/n_{F}}$	Freundlich	Freundlich (1906)	Hg: 0.8796 Pb: 0.881 Cd: 0.8671 As: 0.9618
*K*_F_ and *n*_F_ are Freundlich constant and experimental parameters relating to bioremediation intensity *C*_e_ and *Q*_e_ equilibrium concentration of metals in aqueous solutions and metals in adsorbing equilibrium				
3	$Q_{e}=Q_{\max}\left[K_{L}C_{e}/\left(1+K_{L}C_{e}\right)\right]$	Langmuir	Langmuir (1918)	Hg: 0.9707 Pb: 0.9906 Cd: 0.8905 As: 0.9614
*Q*_e_ and *C*_e_: metal concentration in adsorbing equilibrium and equilibrium concentration in the aqueous phase *Q*_max_ is the maximum amount of the adsorbed metals at high *C*_e_ and *K*_L_ is the Langmuir adsorption constant				

The Langmuir model showed that the biosorption process was homogeneous, uniform, and monolayer, in which inhibitory chemical interactions were detected between the adsorbing molecules. Freundlich model biosorption process was heterogeneous, ununiform, multilayer, and not ideal (Chen et al., 2019), in which suppressing chemical interactions were seen between the adsorbed molecules (Raoov et al., 2013). Similar results about fitted Langmuir isotherm model were reported by Massoud et al. for biosorption of Hg and Cd by *S. cerevisiae* (2019a, b; 2020). Moreover, As biosorption by yeast matched with Freundlich model. The Langmuir fitted model shows a monolayer homogeneous, uniform, biosorption process. In Langmuir model, suppressing chemical interactions were detected between the adsorbing molecules. In contrast, the Freundlich model shows a multilayer, heterogeneous, and nonuniform biosorption process (Chen et al., 2019). In this model, chemical interactions were seen between the adsorbed molecules (Raoov et al., 2013).

## 4 CONCLUSION

The current study investigated the SMG effect on simultaneous bioremediation of four heavy metals in an aqueous solution using *S. cerevisiae* ATCC 9763. The results showed that SMG not only did not have a negative effect on the absorption of tested heavy metals but also a positive effect was observed on the absorption of Hg and Pb. Accordingly, in order to do heavy metal bioremoval using *S. cerevisiae* in the food and beverage industry, SMG treatment can be used to increase efficiency. It should be noted that the metal–yeast complex under GIT exposure was relatively reversible. Therefore, metal–yeast binding is noncovalent and depends on the yeast cell wall structure and the type of metal. According to the metal release from yeast in simulated stomach conditions and reattachment in simulated intestinal exposure, it was found that the attachment of metal–yeast depends on the pH of the environment. Considering the reabsorption of released metals from complex after small intestinal exposure, it can be concluded that this probiotic can be safely used for flight crews and space travelers.

Kinetic and adsorption isotherms models (Temkin, Freundlich, and Langmuir) studies showed that biosorption processes of heavy metal by *S. cerevisiae* cells followed the pseudo‐second‐order kinetic model. Between isotherm models, Langmuir predicts the bioremoval efficiency of Hg, Pb, Cd, and As by *S. cerevisiae*. Both Langmuir's and Freundlich's isotherm models showed precise predictions for As biosorption. Since the biosorption process is still unknown, further research should be conducted to investigate and optimize the heavy metal binding sites on probiotics cell envelopes in microgravity conditions. This study showed that microgravity is a favorable condition for heavy metal bioremoval using *S. cerevisiae*, and yeast–metal complexes are stable enough in gastrointestinal conditions. The result of this investigation may apply to the use of microgravity conditions in heavy metal bioremediation from water and can be applied to the food and beverage industry.

## AUTHOR CONTRIBUTIONS

**Maryam Salavatifar:** Conceptualization (equal); data curation (equal); formal analysis (equal); funding acquisition (equal); investigation (equal); methodology (equal); resources (equal); software (equal); visualization (equal); writing – original draft (equal). **Kianoush Khosravi‐darani:** Conceptualization (equal); data curation (equal); formal analysis (equal); funding acquisition (equal); investigation (equal); methodology (equal); resources (equal); software (equal); visualization (equal); writing – original draft (equal).

## FUNDING INFORMATION

Not applicable.

## CONFLICT OF INTEREST STATEMENT

The authors report no conflicts of interest.

## Data Availability

The data that support the findings of this study are available on request from the corresponding author. The data are not publicly available due to privacy or ethical restrictions.
